# Combination Antifungal Therapy and Formulary Optimization for Progressive Invasive Pulmonary Aspergillosis in a Pediatric Patient With Acute Myeloid Leukemia: A Case Report

**DOI:** 10.1002/ccr3.73041

**Published:** 2026-07-01

**Authors:** Faryal Khamis, Amal Al Busaidi, Kawther Al Bahrani, Asia Al‐Rashdi

**Affiliations:** ^1^ Infectious Disease Unit, Department of Medicine Royal Hospital Muscat Oman; ^2^ Department of Hematology Royal Hospital Muscat Oman; ^3^ School of Medical Sciences University of Manchester Manchester UK

**Keywords:** acute myeloid leukemia, caspofungin, invasive pulmonary aspergillosis, liposomal amphotericin B, pediatrics

## Abstract

Therapeutic response in refractory invasive pulmonary aspergillosis may depend not only on antifungal class escalation, but also on formulation choice. In this complex pediatric acute myeloid leukemia (AML) case, radiologic improvement followed combination therapy and switch to brand‐name liposomal amphotericin B, underscoring the importance of individualized antifungal optimization.

## 1 Introduction

Invasive pulmonary aspergillosis (IPA) is a significant cause of morbidity and mortality in immunocompromised children, particularly those undergoing treatment for acute myeloid leukemia (AML) [1, 2]. The risk is highest during periods of profound and prolonged neutropenia following chemotherapy [3]. Diagnosis is frequently challenging, as clinical signs are non‐specific and blood cultures are rarely positive [4]. Consequently, diagnosis often relies on a combination of host factors, clinical suspicion, radiological findings, and non‐culture‐based microbiological markers [5].

Chest computed tomography (CT) findings, such as the “halo sign,” are highly suggestive of IPA in neutropenic patients but are not specific [6]. The detection of galactomannan (GM), an *Aspergillus* cell‐wall component, in serum or bronchoalveolar lavage (BAL) fluid is a key diagnostic adjunct, especially when cultures are negative [5, 7]. Voriconazole is the recommended first‐line therapy for IPA in children over 2 years old, with liposomal amphotericin B (L‐AmB) as a primary alternative [5, 8]. However, treatment can be complicated by drug toxicity, resistance, or breakthrough infections. This case report describes the successful management of progressive, culture‐negative IPA in a pediatric AML patient, which required escalation to combination antifungal therapy and a formulary switch, illustrating critical decision‐making in a complex clinical scenario.

## 2 Case History/Examination

A previously well 13‐year‐old girl developed stage 4 chronic kidney disease (CKD) in August 2021, presenting with anemia and hypertension. Peritoneal dialysis was initiated in October 2021. In September 2022, she underwent a living‐related renal transplant and was maintained on immunosuppression (prednisolone 20 mg twice daily, tacrolimus 3 mg twice daily, and mycophenolate 1500 mg/day). Postoperatively, she had normal graft function with good urine output and a serum creatinine of 56 μmol/L (reference range 35–75 μmol/L). In November 2023 (Day 1), she presented with several weeks of fever, cough, fatigue, weight loss, and non‐drenching night sweats.

## 3 Differential Diagnosis, Investigations and Treatment

Laboratory tests revealed bicytopenia, with hemoglobin 7.4 g/dL (11–14.5 g/dL) and platelets 41 × 10^9^/L (150–450 × 10^9^/L). The total white blood cell count was 62.7 × 109/L (2.4–9.5 × 10^9^/L), with neutrophils 0.7 × 10^9^/L (1–4.8 × 10^9^/L), lymphocytes 60.1 × 10^9^/L (1.2–3.8 × 10^9^/L), and monocytes 1.8 × 109/L (0.1–1.3 × 10^9^/L), as shown in Table 1. The peripheral blood film showed numerous blast cells, and bone‐marrow examination confirmed AML (Figure 1). Transthoracic echocardiography revealed a heterogeneous right atrial free‐wall mass measuring 12 × 12 mm, which was subsequently confirmed on cardiac magnetic resonance imaging (MRI). The lesion was managed as a right atrial thrombus with anticoagulation under multidisciplinary supervision, with careful consideration of chemotherapy‐associated thrombocytopenia and bleeding risk. No hemodynamic compromise or clinically evident embolic complications were documented.

**TABLE 1:** Baseline post‐transplant and presenting laboratory findings.

Parameter	Value	Reference range
*Post‐transplant graft function*		
Serum creatinine	56 μmol/L	35–75 μmol/L
*At presentation with AML*		
Hemoglobin	7.4 g/dL	11–14.5 g/dL
Platelet count	41 × 10^9^/L	150–450 × 10^9^/L
Total white blood cell count	62.7 × 10^9^/L	2.4–9.5 × 10^9^/L
Neutrophils	0.7 × 10^9^/L	1–4.8 × 10^9^/L
Lymphocytes	60.1 × 10^9^/L	1.2–3.8 × 10^9^/L
Monocytes	1.8 × 10^9^/L	0.1–1.3 × 10^9^/L

*Note:* Data are presented as measured laboratory values with the corresponding institutional reference ranges.

Abbreviations: AML, acute myeloid leukemia; CKD, chronic kidney disease.

**FIGURE 1:**
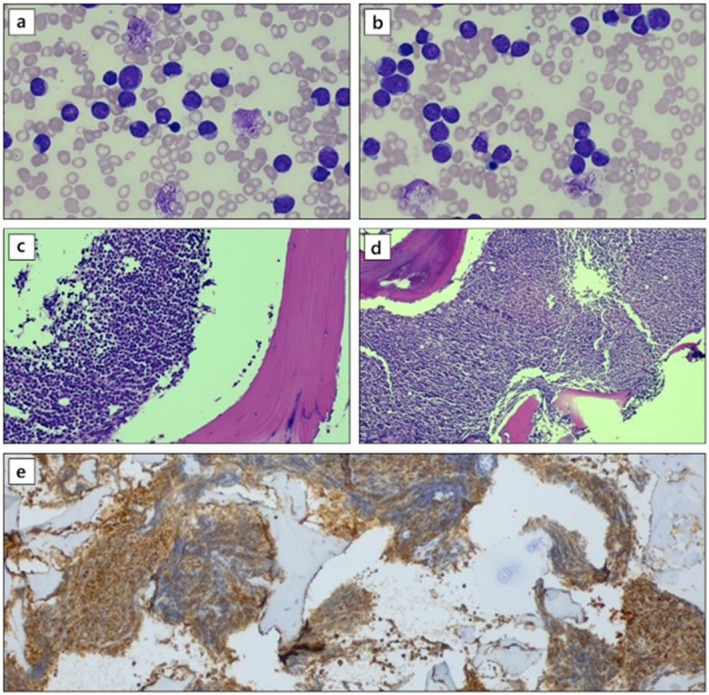
Peripheral blood film and bone marrow pathological findings at presentation with acute myeloid leukemia. (a, b) Peripheral blood film demonstrating numerous circulating blast cells, consistent with acute leukemia. (c, d) Bone marrow examination showing diffuse marrow infiltration by sheets of atypical blast cells, replacing the normal hematopoietic elements. (e) Immunohistochemical staining of the bone marrow biopsy highlighting the blast infiltrate and supporting the diagnosis of acute myeloid leukemia.

She received induction chemotherapy consisting of cytarabine 100 mg/m^2^/day on days 1–10, daunorubicin 30 mg/m^2^/day on days 1–5, and etoposide 100 mg/m^2^/day on days 1–5. Primary mold‐active prophylaxis with voriconazole was administered during induction chemotherapy because of the anticipated high‐risk neutropenic period. Two weeks after completing induction, she developed febrile neutropenia. Empirical piperacillin–tazobactam 4.5 g three times daily was initiated, and blood cultures subsequently grew *Moraxella nonliquefaciens* . She remained persistently febrile (38.4°C–39.0°C) and neutropenic, with a white blood cell count < 0.3 × 10^9^/L, prompting escalation of antibiotics to meropenem 1 g three times daily. A chest CT demonstrated bilateral upper‐lobe consolidations with surrounding ground‐glass “halo” opacities suspicious for invasive mold infection (Figure 2). Flexible bronchoscopy with BAL showed septate fungal hyphae consistent with *Aspergillus species*. The bronchoalveolar lavage galactomannan (BAL GM) index was positive at 0.75 (cut‐off 0.5) and subsequently increased to 6.67, while fungal cultures remained negative. Based on these findings, a diagnosis of probable IPA was made, and antifungal therapy was escalated to treatment‐dose intravenous (IV) voriconazole, with a loading dose of 360 mg followed by 200 mg twice daily, starting on 24 December 2023 and continued until 6 February 2024. Serum therapeutic‐drug monitoring for voriconazole was not available locally; therefore, treatment monitoring was based on clinical response, serial radiological assessment, laboratory surveillance, assessment of neurotoxicity and hepatotoxicity, and review of drug–drug interactions with concomitant immunosuppressive therapy.

**FIGURE 2:**
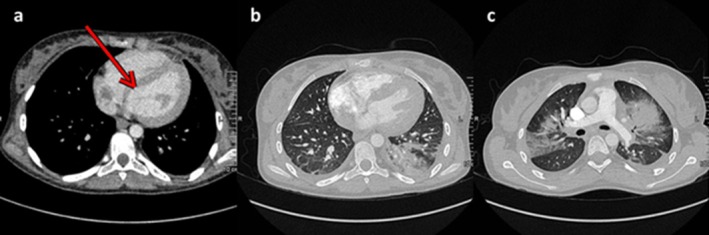
Axial contrast‐enhanced chest CT images. (a) Cardiac‐level image demonstrating a mural filling defect attached to the right atrial free wall (arrow), consistent with a right atrial thrombus after correlation with echocardiography and cardiac MRI. (b, c) Lung‐window images at different axial levels showing bilateral patchy consolidations and ground‐glass opacities with a halo appearance (late 2023).

In January 2024, the patient received salvage FLAG‐IDA chemotherapy (fludarabine, cytarabine [Ara‐C], granulocyte colony‐stimulating factor [G‐CSF] and idarubicin), followed by high‐dose cytarabine (HiDAC); however, bone marrow transplantation was deferred because of the active invasive fungal infection. During salvage chemotherapy, the patient was already receiving treatment‐dose antifungal therapy for probable IPA; therefore, subsequent antifungal management was continued for active infection rather than primary prophylaxis. A follow‐up CT on February 4, 2024, showed some parenchymal improvement with the development of cavitary lesions (Figure 3). However, voriconazole was discontinued on 6 February 2024 because of neurotoxicity, including tremors and severe peripheral neuropathy, as well as significant drug–drug interactions with her immunosuppressive agents. Antifungal therapy was switched to IV L‐AmB at 3 mg/kg (150 mg) once daily.

**FIGURE 3:**
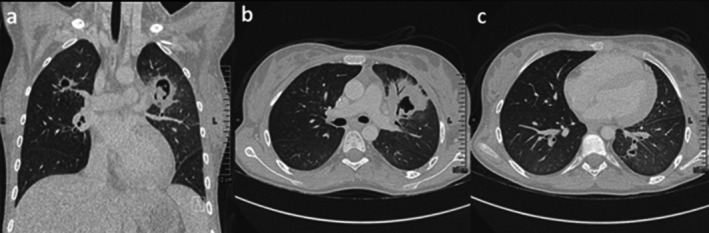
Follow‐up chest CT demonstrating partial parenchymal response with evolving cavitary lesions. Coronal (a) and axial (b, c) lung‐window CT images obtained on 4 February 2024 show interval parenchymal improvement compared with baseline, with reduction of diffuse consolidations but development of new cavitary lesions in the upper lobes and residual patchy opacities (February 2024).

A surveillance CT on 14 April 2024 revealed redemonstration and interval progression of pulmonary lesions (persistent left upper‐lobe cavity, worsening left‐lower‐lobe disease with adjacent ground‐glass change, and enlargement of two basal nodules), as shown in Figure 4. Caspofungin was added on 16 April 2024 (70 mg loading dose followed by 50 mg once daily) but was discontinued on 6 May 2024 because of a drug–drug interaction. Therapy was then switched to anidulafungin (200 mg loading dose followed by 100 mg once daily) from 7 to 21 May 2024, after which it was stopped due to the development of ataxia and vertigo. A repeat bronchoscopy on 28 April 2024 again showed fungal hyphae favoring *Aspergillus* spp. with a positive GM and persistently negative fungal cultures.

**FIGURE 4:**
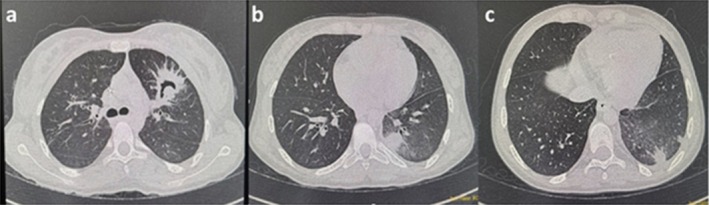
Surveillance CT demonstrating interval progression of pulmonary lesions in invasive pulmonary aspergillosis. Axial lung‐window CT images at different levels: (a) persistent left upper‐lobe cavitary lesion; (b) worsening left lower‐lobe consolidation with adjacent ground‐glass change; and (c) enlargement of two basal pulmonary nodules (April 2024).

Persistent disease activity on combination therapy raised concern for breakthrough aspergillosis and suboptimal amphotericin formulation. Accordingly, on 16 April 2024, treatment was switched from a generic to branded L‐AmB (AmBisome) at a dose of 180 mg (5 mg/kg). Posaconazole 400 mg twice per day was introduced on 26 May 2024 but was stopped on 2 July 2024 owing to QT‐interval prolongation. A follow‐up CT scan on 20 June 2024 demonstrated a radiological response with regression of the cavitary lesions (Figure 5).

**FIGURE 5:**
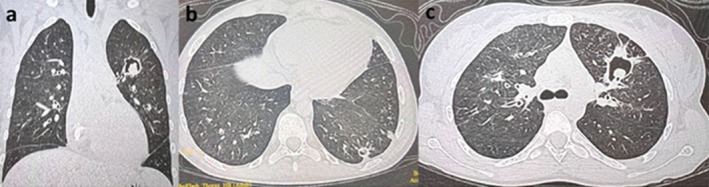
Follow‐up CT demonstrating radiological response with regression of cavitary lesions. Coronal (a) and axial (b, c) lung‐window CT images show marked radiological improvement compared with prior scans, with regression of previously noted cavitary lesions and overall reduction of parenchymal abnormalities (June 2024).

AmBisome was continued until 31 July 2024; the main adverse effect was intermittent hypomagnesaemia, with a nadir of 0.56 mmol/L (reference range 0.66–1.07 mmol/L), which was corrected with supplementation (Table 2 and Figure 6). Isavuconazole 200 mg once daily was then initiated on 31 July 2024 and continued until 21 August 2024. Throughout treatment, renal allograft function was closely monitored, and no dialysis was required.

**TABLE 2:** Clinical timeline from AML diagnosis and evolution of antifungal therapy.

Time from AML diagnosis^a^	Key clinical event/finding	Antifungal regimen
At diagnosis (Month 0)	AML diagnosed; CT chest shows bilateral consolidations with halo sign; BAL GM positive and hyphae seen, suggesting probable IPA	Primary voriconazole prophylaxis during induction chemotherapy; subsequently escalated to treatment‐dose IV voriconazole after probable IPA was diagnosed
1 month	Initiation of first‐line antifungal therapy	Treatment‐dose IV voriconazole continued for probable IPA
2–3 months	Follow‐up CT shows partial improvement with new cavitary lesions; voriconazole discontinued due to neurotoxicity	Switched from voriconazole to IV liposomal amphotericin B (generic L‐AmB)
5 months	Surveillance CT shows radiological progression of pulmonary lesions	Escalation to combination therapy: generic L‐AmB + caspofungin (70 mg loading dose, then 50 mg once daily)
5.5 months	Concern about breakthrough infection and suboptimal formulation	Switched from generic to branded liposomal amphotericin B (AmBisome); caspofungin briefly continued then changed to anidulafungin
6.5–7 months	Additional azole therapy introduced to optimize coverage; CT on 20 June 2024 shows radiological response with regression of cavitary lesions; AmBisome continued	AmBisome continued; posaconazole 400 mg twice daily added
8–9 months	Posaconazole discontinued on 2 July 2024 owing to QT‐interval prolongation; AmBisome continued until 31 July 2024 with sustained radiological response; short consolidation phase with an alternative azole	AmBisome discontinued on 31 July 2024; isavuconazole 200 mg once daily given from 31 July to 21 August 2024
18 months	Ongoing oncological care for AML; no dialysis required; serial cardiac assessment showed interval reduction of the right atrial thrombus during anticoagulation, without hemodynamic or clinically evident embolic sequelae; patient died in May 2025 from causes unrelated to IPA	No ongoing antifungal therapy at the time of death

Abbreviations: AML, acute myeloid leukemia; BAL, bronchoalveolar lavage; CT, computed tomography; GM, galactomannan; IPA, invasive pulmonary aspergillosis; IV, intravenous; L‐AmB, liposomal amphotericin B; QT, QT interval.

^a^ Time intervals are approximate and are expressed relative to the date of AML diagnosis (Month 0).

**FIGURE 6:**
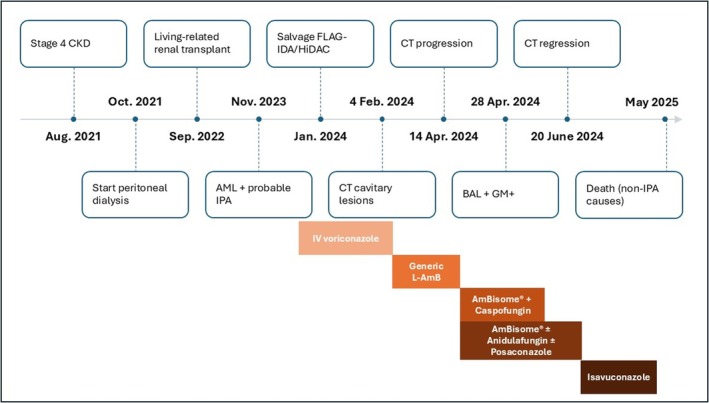
Timeline of key clinical events and antifungal therapy in a renal‐transplant recipient with AML and probable invasive pulmonary aspergillosis. AML, acute myeloid leukemia; BAL, bronchoalveolar lavage; CKD, chronic kidney disease; CT, computed tomography; GM, galactomannan; IPA, invasive pulmonary aspergillosis; L‐AmB, liposomal amphotericin B.

## 4 Conclusion and Results (Outcome and Follow‐Up)

Serial cardiac assessment demonstrated progressive reduction of the right atrial thrombus during anticoagulation, without hemodynamic compromise or clinically evident embolic sequelae. At last follow‐up, the patient remained on antifungal therapy tailored to tolerability and was receiving ongoing oncological care for AML; however, she subsequently died in May 2025 from causes unrelated to IPA.

## 5 Discussion

This case illustrates the complex management of probable IPA in a high‐risk pediatric patient with AML. The diagnosis and treatment pathway highlights several key clinical challenges and educational points. The diagnosis of IPA was established as “probable” according to well‐known criteria [5, 9]. In a profoundly neutropenic patient with AML, the presence of the CT halo sign is a classic early indicator of angioinvasive fungal infection, most commonly IPA [6, 10]. Although not pathognomonic, its appearance prompted urgent further investigation. The diagnostic yield was significantly enhanced by bronchoscopy with BAL. The combination of fungal hyphae on cytology and a highly positive BAL GM index (6.67) provided strong evidence for *Aspergillus* etiology, despite negative fungal cultures [5, 7]. Culture‐negative disease is common, making non‐culture‐based methods indispensable for timely diagnosis and initiation of therapy [11].

Patients with AML receiving induction or salvage chemotherapy represent a particularly high‐risk population for invasive fungal disease because severe chemotherapy‐induced myelosuppression is frequently accompanied by prolonged neutropenia and infectious complications. In this context, radiological and mycological evidence supporting probable IPA should prompt early mold‐active therapy, close radiological and microbiological reassessment, and timely escalation when disease progression is documented or standard therapy is limited by intolerance or drug–drug interactions. Real‐world studies have also supported the feasibility of combination antifungal therapy as a salvage approach for proven or probable invasive fungal disease in selected high‐risk hematological patients, particularly when infection progresses despite monotherapy [12, 13].

Voriconazole is the recommended first‐line agent for IPA in children over 2 years old due to its proven efficacy [5, 14]. However, its use was limited in this patient by neurotoxicity, a known adverse effect that necessitates careful monitoring [15, 16]. The switch to L‐AmB was an appropriate second‐line choice, consistent with international guidelines for cases of intolerance [14, 17].

The radiological progression of disease on L‐AmB monotherapy presented a critical juncture. Escalation to combination therapy with an echinocandin (caspofungin) was a logical next step. While evidence for first‐line combination therapy is debated, it is often considered a salvage strategy for refractory or progressive IPA [18, 19]. The rationale is to target the fungal cell wall (echinocandin) and cell membrane (polyene) for potential synergistic activity [20]. This approach appeared to contribute to the eventual control of the infection in our patient.

The decision to switch from a generic L‐AmB formulation to brand‐name AmBisome coincided with worsening renal function and progressive disease. While this report cannot establish a causal link or difference in efficacy, the choice reflects a critical aspect of antifungal stewardship [21]. In a patient with pre‐existing renal impairment and refractory infection, ensuring optimal drug delivery and minimizing toxicity is paramount. The clinical team's decision was likely driven by a desire to control all variables in a deteriorating situation, prioritizing the use of the formulation with the most extensive clinical data in high‐risk populations. This highlights that stewardship is not merely about cost containment but also about the strategic selection of agents to maximize the probability of a favorable outcome [21].

In high‐risk neutropenic patients with suggestive CT findings, early bronchoscopy with BAL, sent for cytology and GM testing, is pivotal when cultures may remain negative. First‐line triazoles such as voriconazole warrant vigilant toxicity surveillance, including assessment for neurotoxicity and hepatotoxicity, review of drug–drug interactions, electrocardiogram monitoring when indicated, and therapeutic‐drug monitoring where available, particularly in children because of pharmacokinetic variability [17]. In this case, azole therapeutic‐drug monitoring was not available, which limited exposure‐guided confirmation of prophylactic and treatment‐dose triazole adequacy. For progressive IPA despite appropriate monotherapy, escalation to combination therapy, such as L‐AmB with an echinocandin, should be considered as a salvage strategy [18, 19]. These decisions are best embedded within a multidisciplinary antifungal stewardship framework that integrates diagnostics, toxicity monitoring, and formulary optimization to improve outcomes.

Managing IPA in pediatric AML demands a dynamic, individualized approach. This case illustrates successful management of refractory disease through timely diagnosis using imaging and non‐culture‐based biomarkers, stepwise modification of therapy in response to toxicity and disease evolution, and escalation to combination antifungal treatment for salvage indications. Throughout, the application of antifungal stewardship principles enabled rational therapeutic selection and monitoring in a complex host, aligning diagnostics with treatment intensity to optimize efficacy and safety.

## Author Contributions

**Amal Al Busaidi:** writing – review and editing, writing – original draft, investigation, methodology. **Faryal Khamis:** conceptualization, writing – review and editing, writing – original draft, supervision, investigation, data curation, formal analysis, project administration. **Kawther Al Bahrani:** writing – original draft, writing – review and editing, investigation. **Asia Al‐Rashdi:** writing – original draft, writing – review and editing, conceptualization, investigation, visualization.

## Funding

This case report was supported by Gilead Sciences through funding medical writing. The funder had no role in the clinical management of the patient; the collection, analysis, or interpretation of the data; or manuscript drafting or the decision to submit the manuscript for publication.

## Ethics Statement

Ethical approval is waived for single case reports, as they do not constitute “research” involving human subjects under federal regulations, according to the University of Rochester Medical Center Guideline for Determining Human Subject Research (Page 4 of 8, Final v. 01/21/2019).

## Consent

Written informed consent was obtained from the patient's legal guardian(s) for the publication of this case report and any accompanying images. A copy of the written consent is available for review by the Editor‐in‐Chief of this journal. The patient's identity has been protected in accordance with ethical guidelines.

## Conflicts of Interest

The authors declare no conflicts of interest.

## Data Availability

The data that support the findings of this study are available from the corresponding author upon reasonable request.
